# Electron Bio-Imaging Centre (eBIC): the UK national research facility for biological electron microscopy

**DOI:** 10.1107/S2059798317007756

**Published:** 2017-05-31

**Authors:** Daniel K. Clare, C. Alistair Siebert, Corey Hecksel, Christoph Hagen, Valerie Mordhorst, Michael Grange, Alun W. Ashton, Martin A. Walsh, Kay Grünewald, Helen R. Saibil, David I. Stuart, Peijun Zhang

**Affiliations:** a Diamond Light Source, Harwell Science and Innovation Campus, Didcot OX11 0DE, England; b Division of Structural Biology, Wellcome Trust Centre for Human Genetics, University of Oxford, Oxford OX3 7BN, England; c Research Complex at Harwell, Harwell Science and Innovation Campus, Didcot OX11 0FA, England; d Crystallography, Institute for Structural and Molecular Biology, Birkbeck College, Malet Street, London WC1E 7HX, England

**Keywords:** Electron Bio-Imaging Centre, eBIC, cryo-EM, cryo-ET, user facilities

## Abstract

The recent resolution revolution in cryo-EM has led to a massive increase in demand for both time on high-end cryo-electron microscopes and access to cryo-electron microscopy expertise. In anticipation of this demand, eBIC was set up at Diamond Light Source in collaboration with Birkbeck College London and the University of Oxford, and funded by the Wellcome Trust, the UK Medical Research Council (MRC) and the Biotechnology and Biological Sciences Research Council (BBSRC) to provide access to high-end equipment through peer review. eBIC is currently in its start-up phase and began by offering time on a single FEI Titan Krios microscope equipped with the latest generation of direct electron detectors from two manufacturers. Here, the current status and modes of access for potential users of eBIC are outlined. In the first year of operation, 222 d of microscope time were delivered to external research groups, with 95 visits in total, of which 53 were from unique groups. The data collected have generated multiple high- to intermediate-resolution structures (2.8–8 Å), ten of which have been published. A second Krios microscope is now in operation, with two more due to come online in 2017. In the next phase of growth of eBIC, in addition to more microscope time, new data-collection strategies and sample-preparation techniques will be made available to external user groups. Finally, all raw data are archived, and a metadata catalogue and automated pipelines for data analysis are being developed.

## Abstract 2

This paper provides an introduction to the Electron Bio-Imaging Centre (eBIC) at Diamond Light Source: an external user facility established in the UK for high-end cryo-electron microscopy. Details are given of the first year of operation along with highlights and future challenges.

## 1. Introduction to eBIC   

In recent years, cryo-electron microscopy (cryo-EM) has undergone a resolution revolution (Kühlbrandt, 2014 ▸; Egelman, 2016 ▸; Merk *et al.*, 2016 ▸), which has led to a substantial increase in the demand for instrument time and cryo-EM expertise. Cryo-EM groups and facilities around the world have struggled with this increased demand. Coupled with the high cost of buying and maintaining the latest generation of microscopes and detectors, this has created an access problem for a large number of structural biologists. In response to this, a number of centres that provide access to high-end instrumentation and are staffed by expert microscopists (Stuart *et al.*, 2016 ▸; Thompson *et al.*, 2016 ▸) have been opened. Examples of these centres are NeCen in the Netherlands, the Janelia Research Campus of the Howard Hughes Medical Institute and the New York Structural Biology Centre, both in the USA, and the electron Bio-Imaging Centre (eBIC) based at the UK national synchrotron, Diamond Light Source (Saibil *et al.*, 2015 ▸).

The main aim of eBIC is to follow the synchrotron model and provide, free at the point of use, access to state-of-the-art equipment used in biological cryo-EM based on peer review of scientific merit and technical feasibility. Furthermore, eBIC aims to help to drive the cryo-EM field forward through strong in-house research and development. In order to achieve both of these goals, 80% of the available instrument time is provided through the peer-review process, while 20% is reserved for commissioning and in-house research. Another important role for eBIC, which is already beginning to be developed, is to build competence in the user community through training courses and user sessions where eBIC staff provide expertise in grid preparation and optimization. The ultimate aim is not only to enhance the capability of the existing user base but also to make cryo-EM accessible to nonspecialists.

Time on the microscopes at eBIC is obtained *via* three different routes: the first two routes are awarded *via* peer-reviewed proposals and are called Rapid and Block Allocation Group (BAG) access (http://www.diamond.ac.uk/Users.html). The peer-review panel consists of a number of UK-based cryo-EM experts, and every proposal submitted to eBIC is reviewed and scored by at least three panel members. Rapid-access calls take place quarterly, require preliminary cryo-EM data and aim to provide a 48 h microscope session within six weeks of the application deadline. Rapid proposals which are unsuccessful owing to the oversubscription of available microscope time are given feedback and may be put forward for the next application round. BAG calls occur every six months and provide a substantial amount of microscope time to an institute or a collection of users for a two-year period commencing six months from the application deadline. BAGs are reviewed every six months, allowing the amount of time allocated and the number of users on the proposal to be altered. This model is based on one first devised by the the European Synchrotron Radiation Facility (ESRF) and now successfully used for macromolecular crystallography beamlines around the world as it provides user-driven flexible access. A typical eBIC session consists of 48 h of instrument time. Time offered *via* these first two routes comes with travel and subsistence for UK users. For European Union (EU) users travel and subsistence may be currently covered *via* iNext, which is funded through the Horizon 2020 programme of the European Union (http://www.inext-eu.org). The only stipulations for the peer-reviewed access routes are that there must be an intention to publish the results and that eBIC/Diamond Light Source and the funders are acknowledged. The third mode of instrument access is paid, *i.e.* proprietary. A limited amount of time is available *via* this route, which is administered by the industrial liaison office at Diamond Light Source and does not require that the data be published.

eBIC presently has two operational FEI Titan Krios microscopes (Krios I and II), both of which are equipped with direct electron detectors (initially each has both an FEI Falcon II and a Gatan K2 after a Gatan Quantum energy filter). Krios II was only recently added to the user program, so the results detailed below are from Krios I only. In its first year of operation Krios I has over-delivered by 35% with regard to the number of external user days that it was projected to provide, based on the synchrotron-beamline model of approximately 17 d of external user time. In addition to the two Krios microscopes, eBIC will soon have two further microscopes: an FEI Talos Arctica 200 keV TEM and an FEI Scios focused ion beam scanning electron microscope (FIB-SEM). The most effective ways of integrating these machines into the eBIC user programme are currently being established. In one model, the Talos will be available to less experienced cryo-EM users as a project-development tool for the optimization of freezing conditions and initial data-set collection. However, we also intend to explore the extent to which the Talos can be used to screen grids before they are transferred to an eBIC Krios microscope, and to perhaps establish a path to predetermine the best data-collection points from overview maps and transfer these maps, thereby maximizing time for data collection on the high-end Krios instruments. The Scios provides the ability to selectively thin down thick samples (such as eukaryotic cells grown on EM grids) to produce slivers of material of a few hundred nanometres in thickness (termed lamellae; Marko *et al.*, 2006 ▸; Schaffer *et al.*, 2015 ▸). This process is quite time-consuming and so it is likely that, at least in the first instance, access will be through specific calls aimed at enabling high-impact projects, for instance requiring the imaging of processes that occur in thicker regions of cells by cryo-electron tomography (cryo-ET).

## 2. Results from the first year of eBIC   

**Figure 1:**
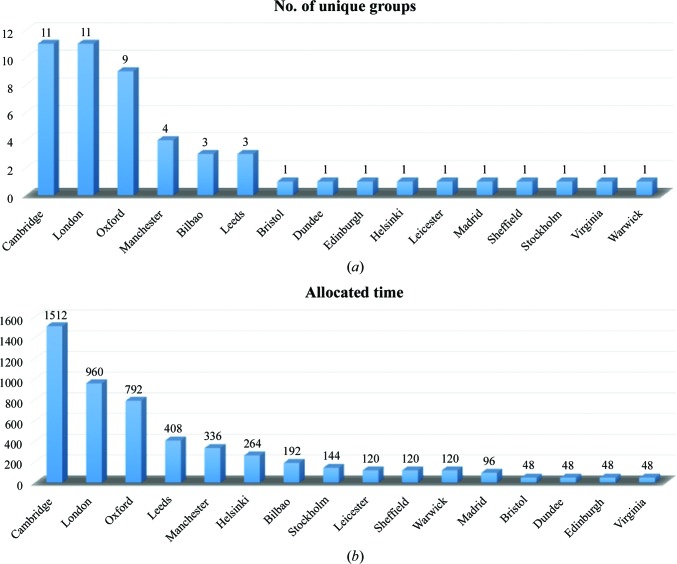
Krios 1 usage. (*a*) A graph showing the total number of unique research groups from different locations that have used Krios 1 during the first year of eBIC. (*b*) The total number of hours for each of these locations. London consists of multiple institutions, including Birbeck College, Imperial College, the Crick Instititute and the Institute of Cancer Research. Cambridge consists of the University of Cambridge and the MRC–LMB.

In its first year of operation, from July 2015 to July 2016, Krios I delivered 222 d to the external user program. This comprised 95 separate visits from 53 different investigators (Fig. 1 ▸). Each visit was either 48 or 72 h in duration, with the first 4–8 h used for sample loading, screening and instrument setup. A breakdown of the time allocated shows that groups from Cambridge, London and Oxford were the largest users of eBIC, which mirrors the distribution of cryo-EM groups in the UK. In addition, groups from both Manchester and Leeds, which also have strong cryo-EM communities, received a significant percentage of the time allocated. At the time of writing of this paper, only Cambridge had direct access to an in-house Krios microscope. However, Cambridge also has the largest in-house community of cryo-EM users. Cryo-EM groups from several countries in continental Europe and the USA have also collected data at eBIC.

The majority of external user sessions at eBIC were collected using the single-particle technique (91%), mainly using the Quantum K2 Summit detector (80%). The Quantum K2 was exclusively used for all external user tomography sessions, as the use of the energy filter is highly desirable for thicker specimens. The effective use of the Quantum K2 detector was facilitated by engagement with the microscope manufacturer (FEI), who agreed to integrate the Quantum K2 with their automated single-particle data-collection software, *EPU*. This integration initially required a few weeks of Krios time but has been broadly successful, although there is still further work required, for instance to routinely use the Volta phase plate (see below). An average 48 h session generates approximately 2 TB of data collected on the Falcon II detector and about 2.9 TB of data collected on the Quantum K2 Summit detector. These volumes of data arise because both detectors collect each projection image as a series of movie frames, and correspond to an average of around 2650 movies on the Falcon II and 2200 movies on the Quantum K2 Summit. The Falcon II collects movies at a somewhat faster rate than the Quantum K2 Summit, as the Falcon II is an integrating detector whilst the Quantum K2 Summit is a counting detector and therefore has a more modest upper limit on the rate at which electrons can be recorded, resulting in longer exposure times. Other factors also affect the data-collection rates, such as the number of frames per movie, the hole size of the grids used and the operation mode of the Quantum K2 Summit (counting *versus* super-resolution). The fastest rates achieved on the Quantum K2 and Falcon II, using equivalent grid types and setups, were 65 and 75 images per hour, respectively.

In total, in the first year of operation Krios I has generated 270 TB of data. The data volumes and rates for eBIC are set to increase with additional microscopes coming online and faster detectors; thus, we expect the data rate to exceed 1 PB per year by the end of 2017. The large volume of data collected highlights another benefit of housing eBIC at a national centre which has the computing resources to handle the storage, transfer and archive of sizeable amounts of data. In practice, this means that as the data are collected on the microscope they are written to Diamond’s central high-speed file system, and once there they are freely available to the user *via* FTP or Globus FTP and are archived to tape almost immediately, where they will be stored for the lifetime of the tape media and will be available for recovery *via* a web interface. This removes the burden of long-term data storage and backup from the host institute of the user. Diamond also has significant computational power (CPU and GPU) tightly coupled to the high-speed file system, so that a significant amount of data analysis can be supported. Work is under way with the data-analysis group at Diamond and with CCP-EM (Wood *et al.*, 2015 ▸) to implement automated pipelines to provide real-time feedback for both single-particle and tomography applications. Underpinning this work will be the effective integration of experimental information management to facilitate data provenance as well as effective experiment tracking, monitoring and eventually integration with data from other disciplines. For this purpose, the *ISPyB* information-management system (Delagenière *et al.*, 2011 ▸) and its interfaces *SynchWeb* (Fisher *et al.*, 2015 ▸) and *SynchLink* (Ginn *et al.*, 2014 ▸), which are already extensively used on macromolecular crystallo­graphy beamlines at Diamond, are being extended to encapsulate eBIC sample tracking as well as single-particle and tomography data collection and processing.

The data collected at eBIC have, at the time of writing, generated ten research publications (Hospenthal *et al.*, 2016 ▸; Serna *et al.*, 2016 ▸; Joseph *et al.*, 2016 ▸; Wilkinson *et al.*, 2016 ▸; Iadanza *et al.*, 2016 ▸; Ramsay *et al.*, 2016 ▸; Fica *et al.*, 2017 ▸; Swuec *et al.*, 2017 ▸; Ilangovan *et al.*, 2017 ▸; Boland *et al.*, 2017 ▸). We have also received a number of personal communications from in-house and external users reporting reconstructions at better than 4 Å resolution, with a few extending beyond 3 Å. The collection of single-particle data sets is routine and if the sample is suitable and the grids are of sufficient quality then high-resolution structures can be expected. The exception to this is for protein complexes smaller than ∼150 kDa, where the phase plate may be needed.

## 3. Future challenges for eBIC   

We anticipate a number of challenges for eBIC and highlight three here in particular.

### 3.1. Working with the Volta phase plate   

One of the main focuses of the in-house research at eBIC has been the incorporation of the phase plate for both single-particle and cryo-ET applications. Without a phase plate, the contrast of an image taken with a modest under-focus will, when Fourier transformed, resemble a sine function, so that there will be little contribution from the crucial low-resolution terms. In comparison, with full phase contrast this function becomes akin to a cosine function, so that contrast is maximized for the low-resolution terms that are critical to locating and orientating small objects in the image, potentially providing a step change in capability. The phase plate currently installed on the Titan Krios microscopes is of the hole-free Volta type (Danev *et al.*, 2014 ▸). This phase plate works *via* the generation of a charge potential on an amorphous carbon film, placed at the back focal plane of the objective lens, that induces a phase shift of the unscattered electrons relative to the scattered electrons. The induced phase shift changes over time, initially increasing rapidly over the so-called conditioning period of the phase plate, followed by a slower increase generating a more stable phase shift. Typically, on our system, using a nominal magnification of 81 000 in EFTEM mode at a dose rate of around 5 electrons per pixel per second (determined on the Quantum K2 Summit), phase-plate conditioning takes around 5 min. Data are then collected during the period of gradual increase in phase shift until the induced phase shift exceeds 90°. In a recent publication the phase plate was changed every hour (approximately 27 images) such that the phase shift did not increase much beyond 90° (Danev & Baumeister, 2016 ▸).

**Figure 2:**
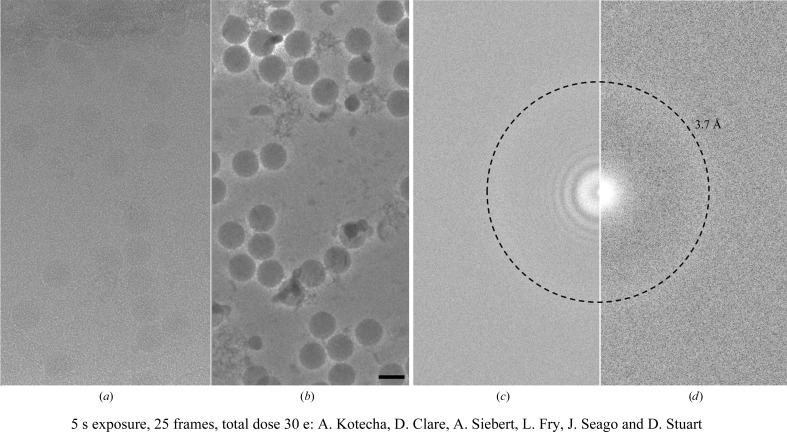
Non-phase-plate *versus* phase-plate data. A cryo-EM micrograph of FMDV taken at 1 µm defocus and its corresponding power spectrum (*a*, *c*) are compared with a micrograph and its corresponding power spectrum when aquired in focus and using the Volta phase plate (*b*, *d*). The images were taken with *EPU* at an equivalent total dose of around 30 e Å^−2^ at a pixel size of 1.06 Å per pixel using Krios 1 at eBIC. Images were collected on the Quantum K2 Summit detector in counting mode (∼6 electrons per pixel per second) with a 20 eV slit width. The FMDV virus particle can be clearly seen in the phase-plate image. The power spectra clearly show that the phase-plate image was in focus as there is no zero CTF present when compared with the 1 µm under-focus power spectrum. The samples were prepared by A. Kotecha, E. E. Fry, J. Seago and D. I. Stuart. The power spectra were calculated using *Bsoft* (Heymann *et al.*, 2008 ▸). The scale bar in (*b*) is 30 nm and the dashed rings in (*c*) and (*d*) are at 3.7 Å resolution.

Both the conditioning time and the period of gradual increase in phase shift can vary for a particular phase plate. The temperature at which the phase plate is maintained in the microscope also has an effect on the characteristics of the phase plate, with higher temperatures increasing the conditioning time required to reach a particular phase shift (Danev *et al.*, 2014 ▸). Another feature of the phase plate is that the quality of the Volta potential is very sensitive to surface contamination, such that there is no guarantee that any two positions on the phase plate will generate equally high-quality phase plates. The effect of this surface contamination can range from mild objective astigmatism to a dramatic distortion of the image. These features make automated data collection with the phase plate more complicated than traditional de­focused imaging. However, even with the increased overheads that the phase plate brings, the large boost in low-resolution contrast, such that defocusing of the objective lens is no longer required, makes it not only advantageous for cryo-ET but also for proteins and complexes, especially those smaller than 150 kDa in mass. The potential of the phase plate for smaller objects is beautifully illustrated by the recent structure of haemoglobin, a 64 kDa protein complex, determined at 3.2 Å resolution (Khoshouei *et al.*, 2016 ▸). In-focus imaging also has the benefit of removing the effects of the contrast-transfer function of the objective lens, potentially making it easier to collect images that are as close to optically perfect as is currently possible (Fig. 2 ▸; Danev & Baumeister, 2016 ▸). However, to achieve resolutions of better than ∼3 Å it is necessary to set the sample focus to −60 nm from absolute focus. The main issue with achieving this level of accuracy is in determining the exact focus of the area of interest. A number of factors make this difficult, in particular the off-area focusing required for low-dose imaging, specimen flatness and tilt. Accurate focus determination is further complicated by the effect of the spherical aberration constant (*C*_s_) of the objective lens, with a recent paper reporting that for a Titan Krios microscope a focus offset of 270 nm was required to accurately set the defocus value to zero. For example, to achieve 20 nm defocus the microscope defocus must be set to 250 nm (Danev & Baumeister, 2016 ▸). This offset is not a constant and depends on the amount of beam tilt that is used for focus estimation. In order to obtain an accurate focus determination, it has been suggested that for single-particle approaches the use of four focus positions around the area of interest is a superior approach to a single focus position. However, this method is very time-consuming and significantly reduces the rate of data collection. Recently, software has been developed that can take the phase shift induced by the phase plate into account during defocus determination, such as *CTFFIND*4 and *Gctf* (Rohou & Grigorieff, 2015 ▸; Zhang, 2016 ▸), and contrast-transfer function (CTF) correction, such as *RELION*2 (Scheres, 2012 ▸). These developments should assist with imaging of objects using a small amount of underfocus (*e.g.* −0.5 µm) and for the correction for this underfocus in the acquired images.

**Figure 3:**
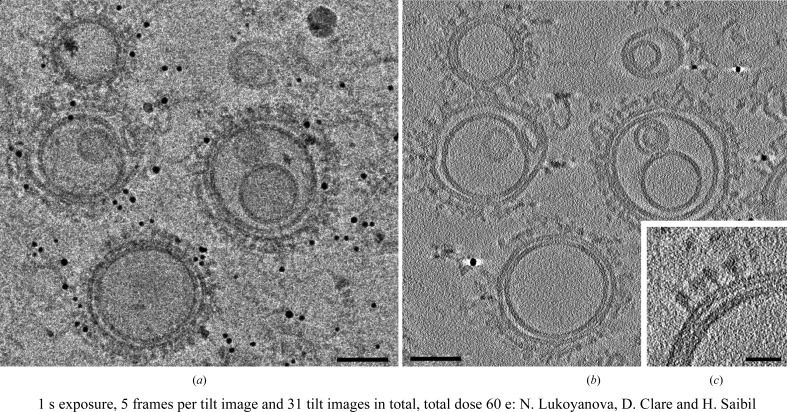
Phase-plate tomography of the perforin pre-pore complex. The 0° image from the tomogram (*a*), the central ten *z* sections averaged from the reconstructed tomogram (*b*) and an enlarged 20-*z*-section average from the reconstructed tomogram (*c*) of perforin pre-pores bound to liposomes collected with the phase plate. The tomogram was collected on Krios II at eBIC at 1.7 Å per pixel using the Quantum K2 detector in counting mode (∼5 electrons per pixel per second) with a 20 eV slit width and a nominal defocus of 300 nm to avoid going over focus. Tilt images were collected from −45 to 45° in 3° increments, giving a total dose of around 60 e Å^−2^. The tomogram was collected using the FEI *TOMO* package and was processed with *MotionCorr* and *IMOD* (Li *et al.*, 2013 ▸; Kremer *et al.*, 1996 ▸). The scale bars in (*a*) and (*b*) are 100 nm and the scale bar in (*c*) is 10 nm. The grids were prepared by N. Lukoyanova.

At the moment the phase plate is not fully integrated into *EPU* and hence new phase plates cannot be generated automatically. The conditioning of new phase plates, approximately every hour, is essential as the phase shift induced by the phase plate increases past 90° in a dose-dependent and time-dependent manner and reduces the quality of the later images. One current solution for this involves the installation of an additional piece of software that moves to the next phase plate at a designated time. For cryo-ET, this is incorporated in the *TOMO* software from FEI, thus making fully automated tomography data collection possible (Fig. 3 ▸). Incorporating the use of the phase plate into the user program is still a work in progress, but its potential for cryo-ET and small single particles will make it highly desirable for many of eBIC’s user groups going forward. We encourage such users to contact the eBIC staff before submitting applications for microscope time.

### 3.2. Sample preparation using FIB-SEM   

**Figure 4:**
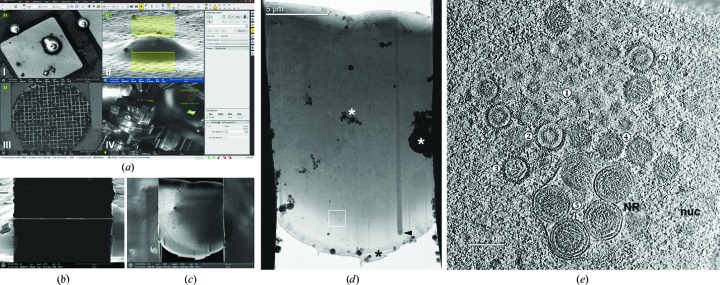
Focused ion beam cryo-milling of herpesvirus-infected cells. Cryo-EM/ET of lamellae produced by focused ion beam (FIB) milling with the eBIC FEI Scios dual-beam scanning electron microscope (SEM). (*a*) Screenshot of the FIB-SEM acquisition software shortly before milling a lamella into a plunge-frozen porcine kidney cell grown on electron-microscopy grids and infected for 10 h with herpesvirus PrVΔUS3 (muliplicity of infection 10). Several imaging modalities support efficient milling, *e.g.* SEM for targeting an appropriate cell specimen (I), FIB imaging for planning and controlling lamella geometry (II), an in-column detector to provide material-specific contrast to check for a protective platinum coat on the sample (III) and an infrared live camera to monitor the cryostage (IV). (*b*) FIB image of the completed lamella through the cell depicted in (*a*) as viewed from the milling angle (18°). (*c*) The same lamella as in (*b*) imaged *via* SEM from the built-in angle of 52° between the electron and ion beams. A low electron acceleration voltage allows the observation of cellular details. (*d*) Low-magnification cryo-EM projection image at 0° of the lamella depicted in (*b*) and (*c*). Before milling, the leading edge was protected from erosion by the gallium ion beam by a platinum layer (black asterisk; ice contamination is shown by white asterisks). Denser objects in the cell led to curtaining (cytoplasmic lipid body; arrowhead). (*e*) Cryo-ET slice of a tomogram taken in the area marked by a white square in (*d*), lamella thickness 130 nm. Visible within the nucleoplasm (nuc) are nucleocapids at different stages of maturation: spherical assemblies of scaffolding protein (1), procapsids (2), partly DNA-filled (3) and nuclear C-capsids (4), which subsequently bud into nucleoplasmic reticulum (NR) forming nuclear egress complex (arrow)-lined capsid-containing vesicles in the perinuclear space (5).

Ideally, biological complexes should be imaged in their native environment inside intact cells. However, TEMs are limited in their penetration power, requiring samples to be less than 0.5–1 µm thick (Koster *et al.*, 1997 ▸). Many groups have coped with this limitation by studying purified biological complexes or thin regions of cells or by using a technique known as cryo-electron microscopy of vitreous sections (CEMOVIS; Al-Amoudi *et al.*, 2004 ▸). While many biological questions can be answered using purified complexes, some interactions, conformations and/or transitional states may not be captured *in vitro*, and many biological complexes and interactions are not present in the thin peripheral regions of cells. While cryo-sectioning is a good alternative, this technique is technically challenging and generates artifacts such as compression of the sample (Al-Amoudi *et al.*, 2005 ▸). The advent of a cryo-capable FIB-SEM (focused ion beam scanning electron microscope), which uses a focused beam of ions (usually gallium) to ablate regions of the sample, has made it possible to thin specific areas of vitrified samples for further imaging by cryo-EM (Marko *et al.*, 2006 ▸; Wang *et al.*, 2012 ▸; Rigort, Bäuerlein *et al.*, 2012 ▸; Rigort & Plitzko, 2015 ▸; Fig. 4 ▸). This technique, coupled with the latest fluorescence cryo-imaging techniques, can provide detailed views of biological processes over a wide range of resolution scales (Rigort, Villa *et al.*, 2012 ▸; Wolff *et al.*, 2016 ▸). Currently, only a small number of laboratories around the world are equipped with cryo-FIB-SEM machines, severely limiting the access of researchers to this technique. At eBIC we will provide both a state-of-the-art instrument (FEI Scios) and the expertise to use it. The user program should start during 2017, giving structural biologists and cell biologists access to this technique using a proposal-based model. As this is currently not a high-throughput technique, only a limited number of collaborative projects will be accepted initially. Success, impact on the community and respective demand will be monitored closely and we encourage potential users to discuss their application with the eBIC staff. Eventually, the intention of eBIC is to offer FIB-SEM coupled with correlative fluorescence microscopy and cryo-ET, providing users with all of the tools necessary to address a wide range of biological questions spanning multiple resolution scales.

### 3.3. Computational requirement   

eBIC already benefits greatly from the computational infrastructure at Diamond, expecially with regards to data-management facilities. If the traditional model is maintained of transferring all of the data to the user’s home laboratory for processing then the current Diamond processes are sufficient, although users will need significant computational and storage resources at their home institution. However, as noted above, we propose to provide close to real-time data-processing pipelines for both single-particle and tomography analyses and to explore the possibility of allowing post-processing of the data. Providing such options will require extensive computational resources. Although eBIC is well placed to benefit and centralize these resources, the current software and data-analysis requirements for processing the data extend well beyond the duration of the measurements. The hardware requirements needed for the processing have also changed markedly over the past 12 months as analysis packages such as *RELION* and *cryoSPARC* (Kimanius *et al.*, 2016 ▸; Punjani *et al.*, 2017 ▸) have provided major accelerations by extensive adaptation to GPU architectures. Together, these developments pose a new challenge and are an area of intensive discussion.

## 4. Conclusions   

In the first year of operation eBIC has delivered free-at-the-point-of-use access to high-end electron microscopy equipment to a large number of different researchers. The number of publications currently stands at ten, but this will increase as the data collected are processed and fully analysed. With further Titan Krios microscopes coming online and the addition of the Talos and Scios instruments, the capacity will increase and more modes of access will be provided. Crucial to future developments will be optimizing the efficient use of the high-end instruments, for example reducing the setup and screening time on the Krios and maximizing data-collection time and data throughput. We will also explore different data-collection software so that we can maximize data throughput and potentially support new data-acquisition schemes. This means that the amount of high-quality data generated at eBIC will significantly increase in the next few years. As well as an increase in the number of microscopes, the incorporation of automated processing pipelines should improve the data quality collected as users will be able to obtain real-time feedback, much like a traditional macromolecular crystallo­graphy beamline at Diamond.
